# Rainbow trout welfare: comparing stunning methods in winter and summer

**DOI:** 10.1007/s10695-025-01526-7

**Published:** 2025-06-09

**Authors:** Roberto González-Garoz, Almudena Cabezas, Montserrat Fernández-Muela, Andrea Martínez Villalba, Elisabeth González de Chávarri, Morris Villarroel, Álvaro De la Llave-Propín, Jesús De la Fuente, Rubén Bermejo-Poza, María Teresa Díaz

**Affiliations:** 1 Animal Production Department, Veterinary Faculty, Complutense University of Madrid (UCM), Avenida Puerta de Hierro S/N, 28040 Madrid, Spain; 2 Department of Animal Science, College of Agricultural Engineering, Technical University of Madrid (UPM), Avenida Puerta de Hierro 2, 28040 Madrid, Spain

**Keywords:** Rainbow trout, Thermal shock, Electric shock, Seasonality, Stress response

## Abstract

This study investigates the effects of three stunning methods: thermal shock (**TS**), electric shock at 200 mA 2 s (**ES2**), and electric shock at 400 mA 0.5 s followed by 200 mA 1.5 s (**ES4**) – both electric shock treatments followed by thermal shock – on the stress response and energy metabolism of rainbow trout (*Oncorhynchus mykiss*) during winter and summer. A significant interaction between stunning method and season was observed for blood cortisol levels. In summer, cortisol concentrations were elevated regardless of the stunning method, whereas in winter, the ES4 method resulted in the lowest cortisol levels. Seasonal variation significantly influenced stress response in trout, with summer conditions leading to higher cortisol levels, darker skin pigmentation, and reduced liver glycogen and lipid reserves. Increased metabolic activity during summer was associated with enhanced reactive oxygen species (ROS) production, which triggered the upregulation of key antioxidant enzymes (*sod*, *gpx*, *cat*, *gst*). The results suggest that the choice of stunning method is critical in mitigating stress, with electrical stunning combined with thermal shock being more effective than thermal shock alone, particularly under elevated temperatures. These findings underscore the importance of optimizing stunning practices to improve fish welfare, especially in the context of climate change. Understanding the interplay between seasonal and procedural stressors provides valuable insights for improving aquaculture management and sustainability.

## Introduction

Rainbow trout (*Oncorhynchus mykiss*) is one of the the most widely farmed finfish species for human consumption, with global aquaculture production reaching 952,691 tons in 2021 (APROMAR 2024). Consequently, a substantial number of fish are slaughtered annually, undergoing various stunning methods that are still unregulated under European legislation. Council Regulation (EC) No 1099/2009, which governs the protection of animals at the time of killing, does not provide specific guidelines on stunning procedures for farmed fish. However, the regulation emphasizes the need for scientific evidence to support recommendations on stunning methods, allowing for the application of different approaches. Furthermore, fish are included under Sect. 1 of Article 3, which mandates that animals should not be subjected to avoidable pain, distress, or suffering. To fulfill this requirement, effective stunning prior to slaughter is essential, ensuring that the procedure is conducted while the fish are in a state of unconsciousness.

Common stunning methods used for rainbow trout include thermal shock through immersion in ice water and electrical stunning, which can be performed under wet or dry conditions (EFSA 2009; HSA 2016). Thermal shock via ice-water immersion is one of the most widely used techniques (Clemente et al. 2023; Saraiva et al. 2024); however, it is generally considered suboptimal due to its association with significant stress and prolonged suffering. This method leads to a slow loss of brain function and delayed unconsciousness, ultimately resulting in death by asphyxiation. Its effectiveness largely depends on the temperature differential between the rearing water and the ice bath, with larger temperature shifts promoting more effective unconsciousness. Consequently, thermal shock is primarily recommended for warm-water species (EFSA 2004; Bordignon et al. 2024). Electrical stunning is generally regarded as a more humane alternative, as it induces immediate unconsciousness while preserving product quality (Saraiva et al. 2024). However, the effectiveness of this method is highly dependent on the correct adjustment of electrical parameters, with current intensity being a key factor in ensuring sufficient duration of unconsciousness (Beyssen et al. 2004). If improperly applied, electrical stunning can result in inadequate or short-lived unconsciousness, compromising animal welfare. Additionally, incorrect settings may cause physical injuries such as bone fractures, muscle damage, and hemorrhages, which not only affect welfare but also diminish product quality (Robb et al. 2002; EFSA 2004). A combination of stunning methods has been proposed as a potential alternative to improve effectiveness and welfare outcomes. Individualized dry electrical stunning, where the electric current is applied to the head, followed by immersion in ice water, has been shown to effectively induce and sustain unconsciousness over time (Sattari et al. 2010).

There is no universal definition of animal welfare but it is generally accepted as the quality of life experienced or perceived by the animal itself. The evaluation of animal welfare is conducted using animal-based or direct indicators and indirect indicators. Direct indicators are measured on the animal and provide an accurate reflection of its physiological state and indirect indicators can help predict potential welfare issues before they become evident through direct indicators, which typically manifest once the animal is already experiencing poor welfare. Direct indicators are related to fish health, growth and behavior, and indirect indicators are related to the environment, rearing practices and handling (Noble et al. 2020). Direct indicators include external parameters, such as skin condition (Noble et al. 2020), where changes in coloration can occur rapidly in response to stress (Vissio et al. 2021), as well as blood parameters, including cortisol levels and biomarkers associated with energy reserve mobilization (Noble et al. 2020). The stress response in fish is a series of neuroendocrine events triggered by a stressful stimulus, aimed at protecting or restoring homeostasis (Johnson et al. 1992). This response progresses through three distinct phases. The primary stress response occurs immediately following exposure to a stressor, involving activation of the sympathetic nervous system and the release of catecholamines. Concurrently, the hypothalamic-pituitary-interrenal (HPI) axis is stimulated, leading to a gradual increase in circulating cortisol levels. This process transitions into the secondary stress response, characterized by the mobilization of energy reserves through the action of catecholamines and cortisol. If the stressor persists, prolonged cortisol exposure can negatively impact multiple physiological systems, including immune function, resulting in stress-induced immunosuppression, a hallmark of the tertiary stress response (Pottinger 2008; Toni et al. 2019). Chronic activation of the stress response has been shown to impair key physiological functions in rainbow trout, including metabolic efficiency, immune performance, and growth potential. Transcriptomic studies have revealed stress-induced alterations in gene expression related to immune mechanisms, energy metabolism, and cell growth and apoptosis, further highlighting the profound effects of stress on trout physiology (Tort 2013).

The mobilization of energy reserves in fish is primarily regulated by cortisol, which influences both lipid and carbohydrate metabolism (Millán-Cubillo et al. 2016). In terms of lipid metabolism, the liver plays a central role as the primary site of lipogenesis and a major lipid storage organ in trout (Sheridan 1989; Tocher 2003). Changes in hepatic lipid content can alter liver morphology, leading to an enlarged size and a shift in coloration from brown-yellow to pale, a characteristic commonly observed in cases of fatty liver disease (Saxena 2017). Lipid mobilization in fish can occur in response to physiological demands such as fasting, reproductive cycles, or migratory behavior, but it can also be stimulated by cortisol (Tocher 2003). During lipid mobilization, triglyceride levels in the bloodstream increase, and as these lipids are metabolized, their levels decline, accompanied by a rise in non-esterified fatty acids (NEFAs) (Sheridan 1988; Alves-Bezerra and Cohen 2017). Carbohydrate metabolism is closely linked to energy homeostasis, with the liver serving as the main glycogen reservoir (Roach 2002). Glucose homeostasis is regulated by the expression and activity of key enzymes involved in glycogenolysis, glycolysis, and gluconeogenesis (Polakof et al. 2012). When energy demands increase, glycogen is degraded via glycogenolysis, generating glucose, which is subsequently utilized through glycolysis. This process is mediated by various enzymes, including hexokinase, which phosphorylates glucose to form glucose-6-phosphate; enolase, which converts phosphoenolpyruvate to 3-phosphoglycerate; and pyruvate kinase, which catalyzes the conversion of phosphoenolpyruvate to pyruvate for entry into the Krebs cycle under aerobic conditions. In anaerobic conditions, pyruvate is instead converted to lactate by lactate dehydrogenase (LDH) (Polakof et al. 2012; Chandel 2021). Following the Krebs cycle, energy-rich molecules such as ATP and GTP, along with oxidizable molecules like NADH and FADH, are generated. These enter the mitochondrial respiratory chain, leading to ATP synthesis. However, this process also results in the formation of reactive oxygen species (ROS), such as superoxide, which can cause significant cellular damage (Rich and Maréchal 2010). The mitochondrial respiratory chain is a primary source of ROS, and while organisms possess antioxidant defense mechanisms to counteract oxidative damage, excessive ROS production can overwhelm these systems, leading to oxidative stress. Key antioxidant enzymes include superoxide dismutase (*sod*), which converts superoxide into hydrogen peroxide; catalase, which further reduces hydrogen peroxide to water; and glutathione peroxidase, which also neutralizes hydrogen peroxide using reduced glutathione, forming oxidized glutathione (Storz and Imlayt 1999; Strange et al. 2001; Margis et al. 2008; Preiser 2012). Oxidized glutathione can subsequently participate in additional detoxification pathways, facilitated by glutathione S-transferase. Gluconeogenesis, the process of glucose synthesis, follows the reverse pathway of glycolysis but requires key enzymes to bypass irreversible glycolytic reactions. Among these, fructose-1,6-bisphosphatase (FBP) plays a crucial role by converting fructose-1,6-bisphosphate into fructose-6-phosphate, ultimately leading to glucose production (Hatting et al. 2018). These metabolic processes collectively regulate energy availability in fish and are influenced by both physiological and environmental factors, including stress and climate change due to rising temperatures.

Seasonal and climate-driven temperature fluctuations can significantly impact the physiological processes in rainbow trout. As poikilothermic organisms, their metabolic rates, oxygen consumption, and enzymatic activity are directly influenced by ambient water temperatures, with a higher metabolic rate at higher temperatures and lower metabolic rate at lower temperatures (Myrick and Cech 2000; Lea et al. 2015; Chang et al. 2020). Rearing rainbow trout at higher temperatures presents additional challenges, as increased water temperatures accelerate metabolic rates. Given that rainbow trout is a cold-water species (Ineno et al. 2005), ongoing climate change and rising water temperatures may significantly impact cold-water fish aquaculture (Van Vliet et al. 2013; Quan et al. 2021). Concern over the impact of increasing temperatures due to climate change on rainbow trout farming has already been reported by Hartman and Porto (2014), who studied the performance of three rainbow trout strains under elevated temperatures. It is also expected that ocean temperature will rise by 1–4ºC in 75 years, affecting fish due to these conditions, and considering that warmer temperatures can enhance the severity of other stressors (Alfonso et al. 2021), this will also affect aquaculture production. These alterations in metabolism and physiology not only affect overall welfare, may also influence the effectiveness of stunning methods prior to slaughter, highlighting the need to assess their impact under variable environmental conditions.

The aim of this study is to investigate the development of the stress response in rainbow trout subjected to thermal shock and two dry electrical shock at two different intensities, both in combination with thermal shock, as stunning methods. This will be assessed through the analysis of cortisol levels, gene expression of glucocorticoid receptors, changes in skin color, metabolic alterations, and the activation of antioxidant mechanisms to mitigate oxidative stress. Additionally, the study seeks to evaluate the impact of seasonality on these responses, considering the poikilothermic nature of fish and the potential influence of environmental temperature on their metabolic processes.

## Materials and methods

### Experimental design

**Fig. 1:**
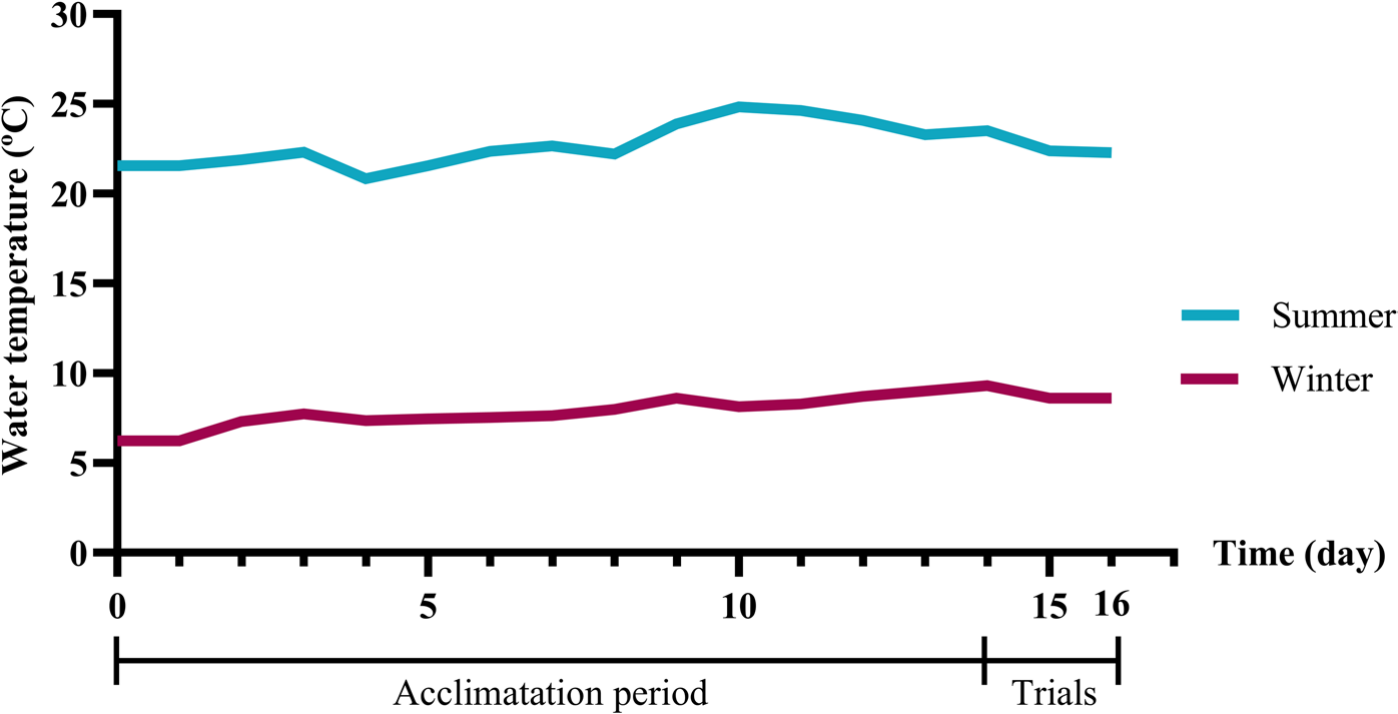
Temperature profile during the acclimation period (14 days) and the trials (2 days) in both seasons studied

Rainbow trout were obtained from the commercial fish farm Cifuentes (Guadalajara, Spain) and transported to the fish farm of the Technical School of Forest Engineering and Natural Environment, Polytechnic University of Madrid (Madrid, Spain). The fish farm used a Recirculating Aquaculture System (RAS) with a water inflow rate ranging from 0.5 to 1L/s and a 20% weekly water renewal rate. Fish were housed in one raceway measuring 6.7 m in length, 1.2 m in width, and 0.65 m in depth, providing a volume of 5.226 m^3^, with a stocking density of 25 kg/m^3^. Dissolved oxygen (8.0 ± 0.3 mg O₂/L) and pH (7.0 ± 0.2) were recorded. Upon arrival at the facility, the fish underwent a two-week acclimation period before being subjected to the trials. Trout were fed a commercial growth diet (EFICO YS 887 F 3, BioMar) at a rate of 1.5% of their body weight once a day at 17:00 h. The experiment was conducted across two seasons: winter (rearing water temperature: 8.67 ± 0.04 °C; photoperiod: 11 h of light and 13 h of darkness) and summer (rearing water temperature: 22.3 ± 0.04 °C; photoperiod: 15 h of light and 9 h of darkness) (Fig. 1). The average fish weight was 349 ± 4.11 g. A total of 180 rainbow trout were sampled, 30 fish per stunning treatment per season.

The stunning treatments employed included thermal shock and two variations of electrical shock. For thermal shock, the fish were immersed in an ice-water mixture at a 1:1 ratio (TS), ensuring the water temperature remained below 2 °C, for 32 min. The dry electrical stunning procedure involved manually restraining the fish and positioning its head between two electrodes to deliver the electrical current directly to the brain. One variation used alternating current at 200 mA for 2 s (90 V, 50 Hz; ES2), while the other applied alternating current at 400 mA for 0.5 s, followed by 200 mA for 1.5 s (90 V, 50 Hz; ES4). Following both electrical stunning methods, the trout were immersed in ice-water for 32 min, which was the same duration as the TS group. The selection of stunning parameters was based on various recommendations. According to EFSA (2004), a temperature of 2ºC is necessary to ensure the effectiveness of thermal shock, leading to unconsciousness in rainbow trout within 9.6 min. Regarding electrical stunning, Robb et al. (2002) suggested that a minimum current of 100 mA at 50 Hz for at least 1 s is required for an effective stun. Also, a current of 400 mA has been shown to be effective for salmon (*Salmo salar*), with longer exposure times needed at frequencies between 50 and 100 Hz.

Prior to stunning, the fish were not subjected to any crowding or fasting. Individuals were randomly selected directly from the holding tank and captured using nets. After capture, each fish was immediately subjected to one of the stunning protocols, alternating between treatments, ensuring that consecutive fish were subjected to different stunning methods. This procedure was followed during two consecutive days in winter and two consecutive days in summer, at the same hour range (10:00–14:00 h). This project was approved by the Ethics Committee of the Technical University of Madrid with reference MDPDSDLPDC-FTB-ANIMALES-20221021.

### Sampling

Immediately after 32 min of ice-water immersion for all the groups, a 1 mL blood sample was collected from the caudal vein and preserved in ethylenediaminetetraacetic acid (EDTA). The sample was then centrifuged at 6000 rpm for 10 min to isolate plasma for the measurement of cortisol, triglycerides, non-esterified fatty acids (NEFAs), glucose, and lactate levels. After blood collection, the animals were sacrificed and eviscerated to obtain the liver. The liver color was recorded, and three liver samples were collected and preserved in liquid nitrogen for further analysis of glycogen reserves, enzymatic activity (including fructose-1,6-Bisphosphatase (FBP), lactate dehydrogenase (LDH), pyruvate kinase (PK), and hexokinase (HK)), and gene expression (Glucocorticoid receptor 1 (*gr1*), mineralocorticoid receptor (*mr*), hypoxia-inducible factor 1 (*hif1*), heat shock proteins 70 and 90 (*hsp70*, *hsp90*), enolase (*eno*), catalase (*cat*), superoxide dismutase (*sod*), glutathione peroxidase (*gpx*), and glutathione S-transferase (*gst*)). Skin color was measured using the CIE Lab* system, with three measurements taken from the dorsal region of the skin (located just behind the dorsal fin on the left-hand side) of each fish at 0 h post-mortem. A Minolta Spectrophotometer CM-2500© (Minolta, Osaka, Japan) was used to capture these measurements, which included the parameters of lightness (L*), redness (a*), and yellowness (b*). From these, chroma (C* = √(a*^2^ + b*^2^)) and hue (h* = arctan(b*/a*) × 57.29) values were calculated, providing insights into the intensity (chroma) and perceived color (hue) of the skin. The same procedure was applied to measure liver color, with three measurements taken from the dorsal side of the liver.

### Laboratory analysis

#### Blood parameters

Cortisol levels were measured using an enzyme immunoassay with a commercial Cortisol ELISA kit (Radim Ibérica S.A., Barcelona, Spain) following manufacturer´s procedure. Triglyceride concentrations were quantified using a fully enzymatic method with a commercial kit from Boehringer Mannheim (Barcelona, Spain). Non-esterified fatty acids (NEFAs) were determined by an enzymatic-colorimetric assay using kits from Randox Diagnostics (London, UK). Glucose and lactate levels in the blood were assessed using enzymatic-spectrophotometric techniques with reagents supplied by Spinreact S.A. (Sant Esteve de Bas, Gerona, Spain).

#### Liver parameters

Liver glycogen reserves were measured using the protocol described by Dreiling et al. (1987). The activity of liver enzymes, including Fructose-1,6-Bisphosphatase (FBP), Lactate Dehydrogenase (LDH), Pyruvate Kinase (PK), and Hexokinase (HK), was assessed through spectrophotometric analysis using a Multiskan GO microplate spectrophotometer (Thermo Scientific, Massachusetts, USA). Enzyme activities were evaluated by monitoring absorbance changes associated with the reduction or oxidation of the coenzymes NADH or NADPH, as outlined by Fernández-Muela et al. (2023).

Liver gene expression was analyzed through quantitative polymerase chain reaction (qPCR). RNA was extracted from liver samples using the Maxwell RSC Simply RNA Tissue kit (Promega, USA), following the manufacturer’s protocol. RNA concentration and purity (A260/A280 ratio) were determined using a Nanodrop 2000 Spectrophotometer (Thermo Fisher Scientific Inc., USA). Complementary DNA (cDNA) was synthesized from 2 μg of RNA using the iScript cDNA Synthesis kit (Bio-Rad, USA), according to the manufacturer’s instructions. Gene expression analysis was conducted via Real-Time quantitative PCR (RT-qPCR) using a CFX Touch™ Real-Time PCR Detection System (Bio-Rad, USA), in compliance with MIQE guidelines (Bustin et al. 2009). The expression of genes related to stress (*gr1*, *mr*, *hif1*, *hsp70*, *hsp90*), oxidative stress (*cat*, *sod*, *gpx*, *gst*), and metabolism (*eno*) was assessed, with *elf1*, *18 s,* and *rps16* serving as reference genes. All pairs of primers had been previously validated in rainbow trout tissues (García-Meilán et al. 2022; Holen et al. 2021; Teles et al. 2013), and their sequences, GenBank accession numbers, efficiency, and product sizes are provided in Table 1. Before conducting the analyses, a dilution curve with a pool of samples was run to determine the appropriate cDNA dilution for each gene to confirm the absence of primer-dimers and the specificity of the reaction by a single peak in the melting curve for each primer set. Reactions were carried out in triplicate wells using 384-well plates with a final volume of 5 μL, containing 2.5 μL iTAq™ Universal SYBR® Green Supermix (Bio-Rad, USA), 0.250 μM of forward and reverse primers, and 1 μL of diluted cDNA per sample. The qPCR program included an initial denaturation step at 95 °C for 3 min, followed by 40 cycles at 95 °C for 10 s and 60 °C for 30 s. The Bio-Rad CFX Maestro 2.3 software was used for data analysis, and the stability of reference genes was confirmed with the geNorm algorithm using Excel. Relative mRNA expression levels were calculated using the 2-ΔΔCT method, with normalization to an arbitrary value of 1 for the TS winter group, which was considered to be under optimal rearing conditions for rainbow trout.

**Table 1:** Sequences of primers used in gene expression analysis and accession numbers

Gene	Official name	Accession number	Sequence 5’ −3’	Efficiency liver (%)	Reference
elf1a	Elongation factor 1	NM_ 001124339	**FW:** TGCCCCTGGACACAGAGATT	104.5	Holen et al. 2021
			**RV:** CCCACACCACCAGCAACAA		
rps16	Ribosomal Protein	tcbk0005c.o.13_5.1.om.4	**FW:** TTTCAGGTGGCGAAACATGC	104.9	Marandel et al. 2015
			**RV:** GGGGTCTGCCATTCACCTTG		
18 s	Ribosomal protein S18	XR_005034822.1	**FW:** TGAGCAATAACAGGTCTGTG	106.9	García-Meilán et al. 2022
			**RV:** GGGCAGGGACTTAATCAA		
hif1	Hypoxia-inducible factor 1 alpha	NM001124288.1	**FW:** TTCTCTGTGCTCTTCTGTGCG	102.1	García-Melián et al. 2022
			**RV:** TGAGTAAGGAAGCAGGGCAA		
gr1	Glucocorticoid receptor 1	Z54210.1	**FW:** CGCAGCAGAACCAACAGTTG	100.6	Teles et al. 2013
			**RV:** ATGAGGGCGTCCAAGTACAGA		
mr	Mineralocorticoid receptor	AF209873.1	**FW:** GGCAGCGTTTGAGGAGATGA	102.0	Teles et al. 2013
			**RV:** CATGGCGTCCAGTAGCTTGG		
hsp70	Heat shock protein 70	NM_001124228.1	**FW:** ATTCTGAACGTAGCAGCGGT	108.0	García-Meilán et al. 2022
			**RV:** GCCATCTTCTCCCTCTGTGC		
hsp90	Heat shock protein 90	AB196457	**FW:** TCCAGCAGCTGAAGGAGTT	99.9	Ings et al. 2011
			**RV:** TGAGCTTGCAGAGGTTCTCA		
eno	Enolase	XM_036988980.1	**FW:** CAAAGGTGTCTCAAAAGCCG	107.8	García-Meilán et al. 2022
			**RV:** GTTGACGTTCTGCCGTACAA		
sod2	Superoxide dismutase	XM_021612540.2	**FW:** TCCCTGACCTGACCTACGAC	103.7	García-Meilán et al. 2022
			**RV:** GGCCTCCTCCATTAAACCTC		
cat	Catalase	XM_021568213.2	**FW:** GCAGTGCCTTTTTGGGTTAGT	96.6	García-Meilán et al. 2022
			**RV:** ACCAAACCACAACTCTTCAGTG		
gpx	Glutathione peroxidase	XM_021569971.2	**FW:** ATTCCCCTCCGATGACTCCA	107.9	García-Meilán et al. 2022
			**RV:** TGGTCAGGAACCTTCTGCTG		
gst	Glutathione-S-transferase	XM_021561454.2	**FW:** TATTGTGGGCTAATGTGTAAGAT	101.0	García-Meilán et al. 2022
			**RV:** CCCTGAAGAGCTTTGTCG		

*FW* Forward primer, *RV* Reverse primer

### Statistical analysis

Statistical analyses were conducted using GraphPad Prism version 10.3.1 (GraphPad Software Inc.). Normality and homogeneity of variance for all parameters were assessed prior to analysis using the Shapiro–Wilk test and Bartlett’s test, respectively. A two-way ANOVA was performed on blood and liver parameters, with the stunning method (TS, ES2, and ES4) and season (winter and summer) as fixed factors. The interaction between these two factors was also evaluated. Post-hoc comparisons of means were carried out using the Tukey test, with statistical significance set at p < 0.05.

## Results and discussion

### Blood cortisol and corticoid receptors gene expression

Plasma cortisol levels are commonly used as an indicator of stress in various fish species, as cortisol is the primary product of the HPI axis, which is activated in response to stress, resulting in elevated blood cortisol levels (Ellis et al. 2012). The measurement of cortisol is particularly valuable for assessing the impact of the slaughter process, which includes stunning and euthanizing the animals. This process can be stressful and painful if stunning is not performed correctly or is ineffective (EFSA 2004; Council Regulation 2009; EFSA 2009).

**Fig. 2:**
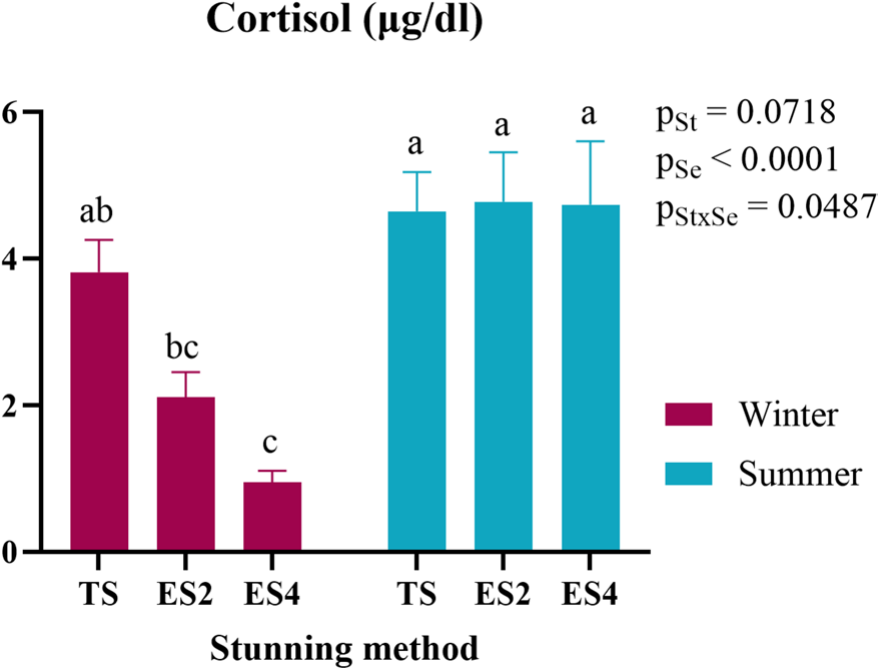
Blood cortisol levels (µg/dl) of rainbow trout stunned by three different methods in summer and winter. St: Stunning; Se: Season; TS: Thermal shock by ice water immersion; ES2: Electric shock at 200 mA for 2 s; ES4: Electric shock at 400 mA for 0.5 s and 200 mA for 1.5 s. Data are presented as mean ± SEM. Different lowercase letters indicate significant differences between groups (p < 0.05)

In the present study, a significant interaction between the stunning method and season was observed in blood cortisol levels (Fig. 2). During the summer, higher cortisol levels were recorded across all stunning methods, with similar levels observed in the thermal shock group during the winter. However, the 400 mA electric shock method in winter resulted in the lowest cortisol concentrations compared to the other groups. The basal level of cortisol for rainbow trout was reported by Alfonso et al. (2023) in 1.7 µg/dl at cold temperature. Villalba et al. (2025) obtained a level of cortisol lower than 1 µg/dl in winter and between 1 and 2 µg/dl in summer in the control group studied in similar conditions. These data support the absence of cortisol release in rainbow trout in ES4 group in winter due to the stunning method and the season, what may suggest the absence of stress response, preserving animal welfare. In summer, all groups have a higher release than the basal level control, indicating a higher stress response due to the stunning method. The elevated cortisol levels observed in the summer can be attributed to seasonality and water temperature, as rainbow trout are cold-water fish with a narrow optimal temperature range, despite tolerating a wide range of temperatures (Chadwick et al. 2015). Alfonso et al. (2021) conducted a literature review which determined that in fish, prolonged exposure to high temperatures can lead to either an increase or a decrease in cortisol levels. When exposure is acute, an increase in cortisol is observed in rainbow trout (*Oncorhynchus mykiss*), whereas prolonged exposure shows a tendency toward an increase. In other salmonids, such as Atlantic salmon (*Salmo salar*) and brook trout (*Salvelinus fontinalis*), the same study highlights a clear increase in cortisol levels when prolongedly exposed to high temperatures. Regardless of the stunning method, trout exhibited the highest cortisol levels in summer. When rearing temperatures are optimal in winter, trout subjected to thermal shock as a stunning method showed cortisol levels comparable to those of summer trout. This may be due to the limited effectiveness of ice-water immersion stunning in cold-water fish (Southgate and Wall 2001; EFSA 2009), although it has been suggested that rainbow trout can lose consciousness with a delay when immersed in a 2 °C ice-water mixture (EFSA 2004). In contrast, electrical stunning is considered an effective method (EFSA 2004, 2009), as demonstrated in salmonids (Lambooij et al. 2010). However, it is crucial to adjust the electric current parameters to ensure proper welfare. For example, in salmon, a minimum current of 100 mA, 50 Hz, and 1 s is required at the brain level (Robb et al. 2002). Similar results have been observed in rainbow trout, where lower cortisol concentrations were recorded when comparing electrical stunning at similar intensities to ice-water immersion at optimal rearing temperatures (Bermejo-Poza et al. 2021) but Saraiva et al., (2024) reported the absence of significant differences between electric stunning al fast chilling (−8ºC).

**Fig. 3:**
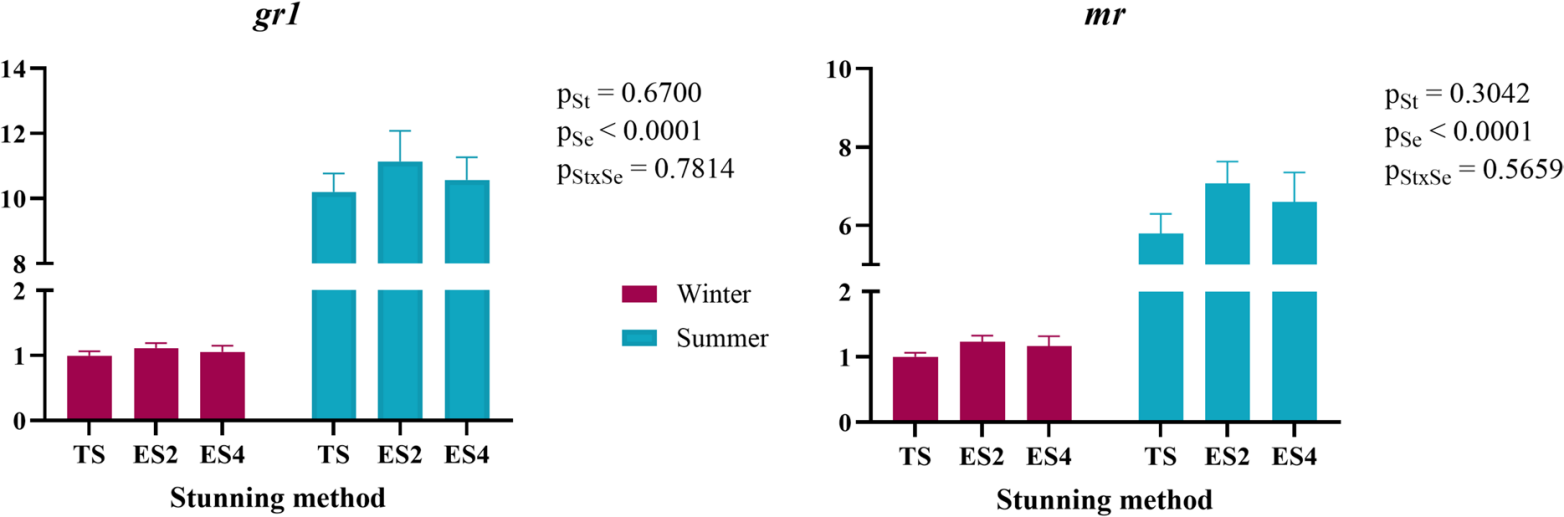
Liver gr1 (glucocorticoid receptor 1) and mr (mineralocorticoid receptor) gene expression of rainbow trout stunned by three different methods in summer and winter. St: Stunning; Se: Season; TS: Thermal shock by ice water immersion; ES2: Electric shock at 200 mA for 2 s; ES4: Electric shock at 400 mA for 0.5 s and 200 mA for 1.5 s. Data are presented as mean ± SEM

The effects of cortisol on an organism, including the regulation of energy metabolism and osmotic balance, are mediated through corticosteroid receptors. In fish, *gr1* and *mr* are among those identified, and investigating the expression of these corticosteroid receptors in the liver is particularly relevant, as the liver plays a central role in energy metabolism and is highly sensitive to environmental changes (Tort 2013; Teles et al. 2013; Quan et al. 2021). The results (Fig. 3) showed that the expression of *gr1* and *mr* receptors was significantly influenced by the season, with higher expression observed in summer compared to winter (10.7 ± 0.43 vs 1.05 ± 0.05 and 6.48 ± 0.35 vs 1.14 ± 0.07, respectively, measured as relative gene expression). The elevated temperatures in summer likely contributed to the increased receptor expression, as they represent a chronic stressor (Quan et al. 2021), further supported by the higher cortisol levels observed in this study during this season. Teles et al. (2013) observed a down-regulation of these receptors during acute stress phases, where cortisol begins to rise, followed by a trend toward upregulation as the situation becomes chronic. Additionally, the results of Benítez-Dorta et al. (2017) in Senegalese sole suggest an up-regulation of *gr1* one hour post-thermal shock in response to increased temperature, which may also explain the observed receptor expression in this study. However, no differences in receptor expression were observed between the stunning methods. Interestingly, although higher cortisol release was detected in the TS group during winter, this was not accompanied by a higher expression of *gr1* or *mr*. This could be explained by the nature of the stunning and slaughter process, which is considered an immediate and rapid procedure (EFSA 2009; Council Regulation 2009).

### Liver gene expression related with hypoxia and thermal shock

Table 2 displays the liver expression results for the *hif1*, *hsp70*, and *hsp90* genes in rainbow trout subjected to thermal shock as a stunning method, compared with rainbow trout stunned by electrical shock at two different current intensities. The use of ice-water immersion as a stunning method in rainbow trout can induce both thermal stress and hypoxia. The sudden change in temperature, along with increased oxygen consumption and potential reduction in oxygen availability, can significantly affect the fish's physiological state (Pörtner and Knust 2007). As the liver is a central organ involved in energy metabolism and stress responses (Teles et al. 2013), it may exhibit specific gene expression alterations in response to these stressors. Hypoxia and thermal shock trigger a series of molecular responses designed to help the fish coping with environments with low oxygen availability and to protect cellular proteins from thermal damage (Wu 2002). Genes involved in acclimatization process, such as hypoxia-inducible factors (HIFs) and heat shock proteins (HSPs), are crucial for the survival and welfare of fish exposed to these challenging conditions (Yamashita et al. 2010; García-Melián et al. 2022).

**Table 2:** Results for the liver gene expression of hif1, hsp70 and hsp90 genes of rainbow trout subjected to different stunning methods in different season

Parameter		TS	ES2	ES4	Mean	p-value		
						Season	Stunning	Se x St
hif1	Winter	1.00 ± 0.08^C^	1.35 ± 0.12^C^	1.20 ± 0.09^C^	1.17 ± 0.06	< 0.0001	0.1010	0.0571
	Summer	16.2 ± 2.47^A^	10.2 ± 1.48^B^	13.2 ± 0.48^AB^	13.3 ± 1.03			
	Mean	8.61 ± 7.61	5.77 ± 4.43	7.21 ± 6.01				
hsp70	Winter	1.00 ± 0.11	1.33 ± 0.29	1.30 ± 0.14	1.22 ± 0.11	0.0341	0.5673	0.5507
	Summer	1.71 ± 0.26	1.90 ± 0.41	1.45 ± 0.22	1.70 ± 0.18			
	Mean	1.36 ± 0.36	1.61 ± 0.29	1.37 ± 0.07				
hsp90	Winter	1.00 ± 0.10	1.19 ± 0.36	1.24 ± 0.15	1.11 ± 0.12	< 0.0001	0.6182	0.6473
	Summer	25.82 ± 3.32	34.0 ± 7.43	35.09 ± 7.01	31.8 ± 3.65			
	Mean	13.4 ± 12.4	17.6 ± 16.4	18.17 ± 16.9				

*hif1* Hypoxia-inducible factor 1, *hsp70* Heat shock protein 70, *hsp90* Heat shock protein 90, *Se* Season, *St* Stunning, *TS* Thermal shock by ice water immersion, *ES2* Electric shock at 200 mA for 2 s, *ES4* Electric shock at 400 mA for 0.5 s and 200 mA for 1.5 s. Data are presented as mean ± SEM. Different uppercase letters indicate differences between groups due to a trend toward an interaction between the season and the stunning method (*p* < 0.1)

For *hif1* gene expression, a seasonal effect has been observed with winter exhibiting lower expression levels compared to summer. Additionally, a trend was observed for the interaction between stunning method and season (p = 0.0571). Regarding seasonality, an enhanced metabolism in summer (Myrick and Cech 2000) may contribute to the observed increase in *hif1* expression driven by secondary stressors such as oxidative stress and cellular damage (Li et al. 2019). The results regarding *hif1* expression could be also linked to hypoxic conditions and may explain the observed interaction trend between stunning and season. However, dissolved oxygen levels were not measured in the ice-water mixture. The lower *hif1* gene expression in winter groups compared to summer groups might be explained by the higher oxygen solubility in cold water (Mehdizadeh and Ashraf 2007) and the decreased metabolic rate of fish in cold water (Myrick and Cech 2000). These factors likely prevent significant oxygen depletion during the 32-min immersion in ice water. The absence of hypoxic conditions—due to both the short exposure duration and high oxygen solubility—along with the lack of enhanced expression under acute hypoxic stress, likely accounts for the lower *hif1* expression in the liver of rainbow trout in winter groups. Han et al. (2022), suggest that acute hypoxia caused by actual production practices such as catching and gathering does not trigger increased expression of *hif1* but decrease in a notable way water dissolved oxygen levels from 5 to 2 mg/L. In contrast, Yang et al. (2017) reported upregulated hif1 levels under acute hypoxia in largemouth bass (*Micropterus salmoides*). In summer, the elevated *hif1* observed could stem from hypoxia induced by the reduced oxygen solubility at higher temperatures (Mehdizadeh and Ashraf 2007) combined with an increased metabolic rate (Myrick and Cech 2000) that depletes oxygen levels in the surrounding water and the aversive scape attempt and due to the lack of effectiveness of thermal shock stunning (EFSA 2004) may have led to higher oxygen consumption, supporting the elevated levels of *hif1* in the TS group in summer compared with the other groups. In the absence of dissolved oxygen measurement, it cannot be confirmed whether hypoxic conditions contributed to the observed gene expression trend.

Marked seasonality was observed in the liver gene expression of *hsp70* and *hsp90*, with higher expression levels recorded in summer compared to winter. Both *hsp70* and *hsp90* are widely recognized as indicators of cellular stress, playing essential roles in protein folding and stabilization, and are activated under conditions such as thermal and oxidative stress (Yamashita et al. 2010). The elevated expression levels observed in this study can be attributed to the impact of high temperatures, which induce thermal stress while simultaneously enhancing oxidative stress due to increased metabolic activity (Myrick and Cech 2000). Similar seasonal variations have been reported by other authors, such as Kuru et al. (2023), who observed greater immunoreactivity to these proteins in the liver of common carp during summer compared to winter. Likewise, studies on rainbow trout have demonstrated higher expression of heat shock proteins under heat stress conditions. For instance, Ma and Luo (2020) and Hosseinpour et al. (2024) found that the acclimation of cold-water species, particularly rainbow trout, to warmer water temperatures resulted in an increase in heat shock protein expression.

### Skin color

The skin functions as a protective barrier and represents the first line of defense, making it a crucial indicator of fish health and welfare (Pavlidis et al. 2006; Noble et al. 2020). Skin color and patterns are primarily determined by chromatophores (Vissio et al. 2021), and can change in response to various stressors (FAO 2014). Such changes are often linked to structural modifications, including the appearance of localized black spots. In rainbow trout, these spots are produced by melanocytes, specialized chromatophores that generate dark eumelanin in response to stressful conditions (Kittilsen et al. 2009). The results for skin color are presented in Table 3.

**Table 3:** Skin color parameters (L*, a*, b*, C*, h) of rainbow trout subjected to different stunning methods in different season

Parameter			TS	ES2	ES4	Mean	p-value		
							Season	Stunning	Se x St
Skin Color	L*	Winter	46.1 ± 1.69	48.7 ± 2.81	47.8 ± 1.06	47.4 ± 1.04	< 0.0001	0.9469	0.8076
		Summer	28.8 ± 3.12	28.0 ± 3.74	27.9 ± 3.05	28.3 ± 1.88			
		Mean	37.4 ± 8.67	38.35 ± 10.3	37.8 ± 9.94				
	a*	Winter	0.70 ± 0.30	0.13 ± 0.30	1.26 ± 0.27	1.06 ± 0.17	0.4826	0.1076	0.7130
		Summer	0.77 ± 0.14	1.15 ± 0.22	0.93 ± 0.24	0.95 ± 0.11			
		Mean	0.74 ± 0.04	1.25 ± 0.09	1.09 ± 0.16				
	b*	Winter	6.45 ± 0.59	6.74 ± 1.09	5.91 ± 0.38	6.34 ± 0.38	0.7968	0.9179	0.7980
		Summer	5.91 ± 0.69	6.24 ± 1.09	6.39 ± 1.06	6.18 ± 0.55			
		Mean	6.18 ± 0.27	6.49 ± 0.25	6.15 ± 0.24				
	C*	Winter	6.75 ± 0.55	7.07 ± 1.05	6.30 ± 0.37	6.67 ± 0.36	0.9998	0.7491	0.6934
		Summer	6.07 ± 0.67	7.04 ± 1.04	7.00 ± 1.02	6.69 ± 0.53			
		Mean	6.41 ± 0.34	7.06 ± 0.01	6.65 ± 0.35				
	h(º)	Winter	74.1 ± 2.50^ab^	71.9 ± 3.68^ab^	73.9 ± 2.30^ab^	73.5 ± 1.57	0.0931	0.0210	0.0468
		Summer	79.3 ± 1.44^a^	62.82 ± 4.74^b^	62.2 ± 5.22^b^	68.0 ± 2.56			
		Mean	76.7 ± 2.61	67.4 ± 4.55	68.1 ± 5.90				

L* Lightness, a* Redness, b* Yellowness, C* $Chroma =\sqrt{{\left({a}^{*}\right)}^{2}+{\left({b}^{*}\right)}^{2}}$, *h (º) *$Hue=\mathit{arctan}\left(\frac{{b}^{*}}{{a}^{*}}\right)$*, Se* Season, *St* Stunning, *TS* Thermal shock by ice water immersion, *ES2* Electric shock at 200 mA for 2 s, *ES4* Electric shock at 400 mA for 0.5 s and 200 mA for 1.5 s. Data are presented as mean ± SEM. Different lowercase letters indicate significant differences between groups (*p* < 0.05)

A seasonal influence on skin lightness (L*) was observed, with darker skin in summer compared to winter. This could be attributed to a photoprotective mechanism, as suggested by Leclercq et al. (2010), since there were more daylight hours during the summer, and skin darkening may help protect against increased solar radiation. Additionally, skin darkening can occur under stressful conditions (Kittilsen et al. 2009), which may explain the darker skin observed in this study, as rainbow trout are a cold-water species and the rearing temperature during summer was above their optimal range (Ineno et al. 2005). This stress response has also been noted in rainbow trout under different stressors, such as fasting (Villalba et al. 2025). Furthermore, an interaction between the stunning method and season was observed in relation to skin hue (hº), with values clustering around a 70º angle, corresponding to a yellow color, in winter. This is likely due to the yellow/golden color of the trout, interspersed with black spots. According to Colihueque et al. (2011), yellow is one of the most common skin colors in rainbow trout, alongside blue and green. In summer, differences in hue were found between the TS group and the ES2 and ES4 groups, with higher values observed in the TS group. A higher hue has been associated with increased stress levels in salmon (Erikson and Misimi 2008), which may explain the greater hue values in the TS group. This suggests that thermal shock may be less effective in rainbow trout, while electrical shock is considered more effective (EFSA 2004, 2009; HSA 2016; Saraiva et al. 2024). Additionally, Sattari et al. (2010) support the effectiveness of electrical stunning followed by ice-water immersion, which may be more efficient in summer due to the greater thermal shock after electrical stunning. The temperature change, a key factor in thermal shock (Zampacavallo et al. 2015), may account for the difference in hue observed in the TS group compared to the ES2 and ES4 groups in summer.

### Energy metabolism

The stress response in animals, including rainbow trout, is closely associated with energy metabolism, as it involves the release of hormones such as adrenaline and cortisol to help the organism cope with physical and psychological challenges. These hormones activate processes like gluconeogenesis and lipolysis, ensuring that energy resources are mobilized to effectively respond to the stressor (Noble et al. 2020).

### Lipid metabolism parameters

Seasonality influenced the mobilization of lipid reserves, as reflected in Table 4. Higher blood triglyceride concentrations were observed in winter compared to summer. Regarding non-esterified fatty acids (NEFAs), an interaction between season and stunning method was found, with significant differences observed between groups stunned in winter and those stunned in summer. In summer, NEFA concentrations were higher. The decrease in triglycerides during the summer and the concurrent increase in NEFAs suggest greater mobilization of lipid energy reserves, as triglyceride consumption leads to higher plasma free fatty acids (Sheridan 1989; Toni et al. 2019). This enhanced mobilization may be attributed to the activation of the hypothalamic-pituitary-interrenal axis in response to increased stress, a characteristic response of cold-water species exposed to elevated water temperatures (Wagner et al. 1997; Chadwick et al. 2015), in addition to a faster metabolism driven by the high temperatures of the summer season, as rainbow trout are poikilothermic organisms (Myrick and Cech 2000). Also, the enhanced mobilization can be attributed to the physiological status associated with higher temperatures, which accelerate metabolic rates and physiological processes, such as increasing development times and growth, and also influence behavior, such as increasing swimming activities (Alfonso et al. 2021). In common carp (*Cyprinus carpio*), it has been reported that liver metabolism is enhanced under temperature stress (Sun et al. 2019). Villalba et al. (2025) in similar climatic conditions reported a basal level of triglycerides and NEFAs in blood in the control group used: triglycerides levels in winter between 150–200 mg/dl and in summer between 100–150 mg/dl and NEFAs between 0.0–0.5 mmol/l in winter and 0.5–1.0 mmol/l in summer. This suggests a basal response of the animals in winter and summer regarding triglycerides levels, which can be explained by the physiological status of the individuals in summer.

**Table 4:** Parameters related with lipid metabolism, including blood parameters (triglycerides and NEFAs) and liver color (L*, a*, b*, C*, h*) of rainbow trout subjected to different stunning methods in different season

Parameter			TS	ES2	ES4	Mean	p-value		
							Season	Stunning	Se x St
Triglycerides (mg/dl)		Winter	222 ± 14.6	237 ± 14.2	222 ± 15.2	227 ± 8.38	< 0.0001	0.8607	0.6384
		Summer	146 ± 9.86	139 ± 7.90	141 ± 8.24	142 ± 5.01			
		Mean	184 ± 38.1	188 ± 48.8	181 ± 40.5				
NEFAs (mmol/l)		Winter	0.19 ± 0.02^b^	0.12 ± 0.01^b^	0.12 ± 0.02^b^	0.14 ± 0.01	< 0.0001	0.5419	0.0290
		Summer	0.32 ± 0.02^a^	0.35 ± 0.03^a^	0.35 ± 0.02^a^	0.34 ± 0.01			
		Mean	0.25 ± 0.07	0.24 ± 0.11	0.24 ± 0.12				
Liver Color	L*	Winter	26.9 ± 0.42^abc^	27.6 ± 0.50^ab^	28.9 ± 0.62^a^	27.8 ± 0.31	< 0.0001	0.2471	0.011
		Summer	23.9 ± 1.28^bc^	22.9 ± 1.45^c^	19.0 ± 1.01^d^	21.9 ± 0.75			
		Mean	25.4 ± 1.45	25.3 ± 2.32	23.9 ± 4.95				
	a*	Winter	9.26 ± 0.41^bcd^	8.88 ± 0.30^ cd^	8.20 ± 0.29^d^	8.78 ± 0.20	< 0.0001	0.0439	0.0002
		Summer	11.6 ± 0.79^b^	11.2 ± 0.74^bc^	14.8 ± 0.81^a^	12.6 ± 0.48			
		Mean	10.4 ± 1.17	10.1 ± 1.18	11.5 ± 3.30				
	b*	Winter	13.3 ± 0.47	13.4 ± 0.41	13.3 ± 0.45	13.4 ± 0.25	0.0884	0.3964	0.3779
		Summer	15.3 ± 0.86	14.1 ± 0.83	13.5 ± 0.77	14.3 ± 0.47			
		Mean	14.3 ± 0.96	13.7 ± 0.37	13.4 ± 0.06				
	C*	Winter	16.4 ± 0.44	16.2 ± 0.39	15.8 ± 0.36	16.2 ± 0.23	< 0.0001	0.8801	0.5058
		Summer	20.0 ± 0.80	20.0 ± 0.94	20.6 ± 0.67	20.1 ± 0.46			
		Mean	18.2 ± 1.76	17.9 ± 1.75	18.2 ± 2.40				
	hº	Winter	54.6 ± 1.31^ab^	56.4 ± 1.23^ab^	58.0 ± 1.48^a^	56.3 ± 0.78	< 0.0001	0.3390	0.0104
		Summer	51.8 ± 2.55^ab^	49.2 ± 2.62^bc^	42.9 ± 2.56^c^	50.0 ± 1.53			
		Mean	53.2 ± 1.36	52.8 ± 3.62	50.4 ± 7.54				

*NEFAs* Non esterified fatty acids, L* Lightness, a* Redness, b* Yellowness, C* $Chroma =\sqrt{{\left({a}^{*}\right)}^{2}+{\left({b}^{*}\right)}^{2}}$, *h*º $Hue=\mathit{arctan}\left(\frac{{b}^{*}}{{a}^{*}}\right)$*, Se* Season, *St* Stunning, *TS* Thermal shock by ice water immersion, *ES2* Electric shock at 200 mA for 2 s, *ES4* Electric shock at 400 mA for 0.5 s and 200 mA for 1.5 s. Data are presented as mean ± SEM. Different lowercase letters indicate significant differences between groups (*p* < 0.05)

Liver color parameters (Table 4) revealed an interaction between stunning method and season. For lightness (L*), no significant differences were observed in the thermal shock stunning group between seasons, while significant differences were found in the electric shock groups. Specifically, in summer, the ES4 group exhibited the lowest luminosity, while in winter, the same group showed the highest luminosity. The redness index (a*) also demonstrated an interaction between stunning method and season, with the ES4 group in summer displaying the highest value. Chroma (C*) reflected significant seasonal effects, with higher values in summer than in winter. Liver hue (hº) again revealed the interaction between stunning method and season, with the lowest hue angle observed in the high-intensity electrical shock group. These findings suggest that winter stunning resulted in a liver with a reddish-orange color that was brighter and less saturated compared to summer. In contrast, summer liver coloration is redder, darker, and more saturated. Seasonal differences may be attributed to the increased mobilization of lipid reserves in the liver during summer, as reflected in triglyceride and NEFA concentrations. A higher amount of lipid reserves contributes to a lighter, more yellowish liver tone, which is particularly evident in conditions such as fatty liver disease, as seen in other species like chickens and ducks (Trampel et al. 2005; Bonnefont et al. 2019). In the case of the ES4 group during summer, the liver appeared darker and redder, likely due to greater hepatic congestion caused by ventricular fibrillation induced by electrical stunning, which impairs venous return. Similar findings have been reported in pigs subjected to electrical stunning (Marcon et al. 2019). This effect may be more pronounced in summer compared to winter due to the reduced lipid reserves, as these contribute to a more yellowish color, making the red less noticeable (Saxena 2017).

### Carbohydrate metabolism parameters

Seasonal effects on carbohydrate metabolism parameters (Table 5) were observed, with lower hepatic glycogen concentrations in summer compared to winter. This difference likely reflects an increased consumption of glycogen during the warmer months, driven by various metabolic adaptations related to temperature and energy demands. In summer, higher temperatures and increased physical activity accelerate metabolic rates, leading to greater reliance on glycogen as an energy source (Myrick and Cech 2000; Lea et al. 2015; Alfonso et al. 2021). Such temperature-induced effects on carbohydrate metabolism have been documented in other species, such as Chinese crucian carp (Yang et al. 2015), and during chronic stress conditions in sturgeon (Lankford et al. 2005).

**Table 5:** Results related with carbohydrate metabolism, including energy reserves, blood parameters, enzymatic activity and gene expression of rainbow trout subjected to different stunning methods in different season

Parameter		TS	ES2	ES4	Mean	p-value		
						Season	Stunning	Se x St
Hepatic glycogen (mg/g)	Winter	164 ± 9.22	149 ± 7.88	148 ± 8.31	154 ± 4.91	< 0.0001	0.3897	0.3585
	Summer	15.2 ± 2.55	13.6 ± 2.08	17.2 ± 2.71	15.34 ± 1.41			
	Mean	90.0 ± 74.8	81.4 ± 67.8	82.7 ± 65.5				
Blood glucose (mg/dl)	Winter	89.6 ± 2.53^A^	87.0 ± 2.64^A^	88.5 ± 2.14^A^	88.35 ± 1.41	< 0.0001	0.0061	0.0546
	Summer	83.5 ± 3.29^A^	67.9 ± 3.10^B^	71.3 ± 3.28^B^	74.58 ± 1.99			
	Mean	86.5 ± 3.06^a^	77.5 ± 9.56^b^	79.9 ± 8.60^ab^				
Liver HK activity (mU/mg)	Winter	1.97 ± 0.24	1.96 ± 0.028	1.66 ± 0.27	1.87 ± 0.15	< 0.0001	0.6657	0.6568
	Summer	0.02 ± 0.00	0.01 ± 0.00	0.02 ± 0.00	0.01 ± 0.00			
	Mean	0.99 ± 0.098	0.98 ± 0.97	0.84 ± 0.82				
Liver FBP activity (mU/mg)	Winter	0.27 ± 0.07	0.31 ± 0.07	0.24 ± 0.06	0.27 ± 0.04	< 0.0001	0.9495	0.7266
	Summer	0.64 ± 0.06	0.59 ± 0.07	0.62 ± 0.05	0.62 ± 0.04			
	Mean	0.45 ± 0.18	0.45 ± 0.14	0.43 ± 0.19				
*enolase* gene expression	Winter	1.00 ± 0.09	1.44 ± 0.24	1.33 ± 0.14	1.24 ± 0.09	< 0.0001	0.6352	0.6211
	Summer	22.0 ± 1.87	22.5 ± 1.87	20.2 ± 0.43	21.7 ± 0.96			
	Mean	11.5 ± 10.5	12.0 ± 10.5	10.8 ± 9.42				
Liver PK activity (mU/mg)	Winter	0.07 ± 0.01	0.06 ± 0.01	0.09 ± 0.02	0.07 ± 0.01	0.0020	0.0709	0.9092
	Summer	0.10 ± 0.01	0.10 ± 0.01	0.13 ± 0.02	0.11 ± 0.01			
	Mean	0.08 ± 0.02	0.08 ± 0.02	0.11 ± 0.02				
Liver LDH activity (mU/mg)	Winter	0.06 ± 0.01^bc^	0.07 ± 0.01^b^	0.14 ± 0.02^a^	0.09 ± 0.01			
	Summer	0.01 ± 0.00^c^	0.02 ± 0.01^bc^	0.02 ± 0.00^bc^	0.02 ± 0.00	< 0.0001	0.0020	0.0080
	Mean	0.04 ± 0.02	0.05 ± 0.02	0.08 ± 0.06				
Blood Lactate (mmol/l)	Winter	5.69 ± 0.27	6.31 ± 0.18	6.35 ± 0.23	6.11 ± 0.14	0.9216	0.6795	0.0084
	Summer	6.57 ± 0.32	5.72 ± 0.24	6.11 ± 0.19	6.15 ± 0.16			
	Mean	6.13 ± 0.44	6.01 ± 0.30	6.23 ± 0.11				

*HK* hexokinase, *FBP* fructose-1,6-Bisphosphatase, *PK* pyruvate kinase, *LDH* lactate dehydrogenase, *Se* Season, *St* Stunning, *TS* Thermal shock by ice water immersion, *ES2* Electric shock at 200 mA for 2 s, *ES4* Electric shock at 400 mA for 0.5 s and 200 mA for 1.5 s. Data are presented as mean ± SEM. Different lowercase letters indicate significant differences between groups (*p* < 0.05). Different uppercase letters indicate differences between groups due to a trend toward an interaction between the season and the stunning method (*p* < 0.1)

Glucose levels in the bloodstream (Table 5) were influenced by both stunning system and season, with differences observed between summer and winter, likely due to the temperature effects on metabolism. During warmer periods, metabolism accelerates, leading to increased glucose consumption and consequently lower glucose levels in the bloodstream. Studies on glucose levels under high temperatures show that while cortisol release may initially raise glucose levels, prolonged exposure to heat often results in a reduction in glucose concentrations. This reduction is attributed to higher energy demands, depletion of carbohydrate reserves, and metabolic disruption during heat stress. For example, in Nile tilapia, glucose levels have been observed to decrease under prolonged exposure to high temperatures (Islam et al. 2020). Basal levels of glucose in control groups in similar climatic conditions were reported in 70 mg/dl in winter and summer, without differences between seasons (Villalba et al. 2025). Other studies underscore the impact of heat stress on glucose utilization and energy dynamics, highlighting the altered metabolisms at high temperatures (Jiang et al. 2022; Lin and Meegaskumbura et al. 2024). Regarding the effect of the stunning method, the higher glucose levels were observed in the TS group, which can be explained by the lower effectiveness of thermal shock in inducing unconsciousness, thereby activating the stress response and the glucose mobilization (HSA 2016; Noble et al. 2020). This explanation is also supported by the trend toward an interaction between the season and the stunning method. In case of ES2 and ES4 groups during summer, lower glucose levels were observed compared to TS group in summer and TS, ES2 and ES4 groups in winter with a tendency between stunning method and season (p = 0.0546). This may be attributed to a reduced stress response from the combined use of electrical shock and thermal shock, which is highly effective in inducing unconsciousness (Sattari et al. 2010). The efficacy of thermal shock depends on the extent of the temperature change, with larger changes being more effective (Zampacavallo et al. 2015). This could explain the lower glucose levels in the ES2 and ES4 groups during summer, as they may be experiencing lower stress due to greater thermal shock after electric shock. The higher levels of TS group in summer could be explained by this greater thermal shock in a consciousness state and the higher glucose levels in TS, ES2 and ES4 groups in winter can be explained by a lower mobilization of energy reserves during this season, maintaining glucose levels.

Regarding hepatic glucose consumption, the enzyme hexokinase (Table 5) exhibited lower activity in summer than in winter. This reduction in activity during warmer months has also been observed in muscle tissues, as seen in goldfish (dos Santos et al. 2010) and lenok (*Brachymystax lenok*) subjected to 48 h of heat stress (Chen et al. 2023). Other glucose-related parameters, such as the expression of *enolase* and the enzymatic activity of pyruvate kinase (Table 5), were higher in summer compared to winter. Ma et al. (2024) reported in the liver a higher activity of the pyruvate kinase enzyme in rainbow trout subjected to chronic heat stress. Similarly, fructose-1,6-bisphosphatase activity (Table 5), involved in glucose formation, was higher in summer, likely due to reduced hepatic glycogen reserves and increased energy demands driven by accelerated metabolism. Consequently, glucose production pathways were activated during the summer months. An increase in FBP activity under high-temperature conditions has been observed in the Patagonian blenny (Oyarzún et al. 2017). The activity of lactate dehydrogenase (LDH) at the hepatic level (Table 5) showed an interaction between season and stunning method, with the highest enzyme activity observed in winter in the ES4 group. Lactate levels (Table 5), resulting from carbohydrate consumption, demonstrated an interaction between season and stunning method, although no statistically significant differences were detected between groups. Thermal shock by immersion in ice water induces strong escape attempts in conscious individuals (Zampacavallo et al. 2015; HSA 2016), with more intense responses during larger temperature changes (summer) and less intense responses during smaller temperature changes (winter). Electric stunning has also been shown to induce muscle effects during the stunning process (HSA 2016), which may explain the observed results in LDH activity and blood lactate levels. In winter, the higher LDH activity in the 400 mA ES4 group could be due to greater muscle stimulation and energy demand compared to the 200 mA stimulation. The intensified escape response in the TS group, combined with higher metabolic rates, may explain the increased enzyme activity. In summer, the lower glucose levels in the bloodstream may reduce LDH enzyme activity, as the reduced glucose availability limits anaerobic metabolism and lactate production. The rapid nature of electrical stunning, coupled with lower glucose levels in summer, might explain the lack of statistically significant differences in blood lactate levels, with the intense escape response from thermal shock accounting for higher lactate production in that group during the warmer season. Villalba et al. (2025) reported blood lactate levels around 3 mmol/l in both seasons, summer and winter, in a control group at similar climatic conditions. The absence of a statistically significant difference in lactate levels may also be attributed to physiological mechanisms activated within the organism to prevent lactate accumulation, thereby avoiding tissue acidosis and compensating for energy deficits. Have been reported the sequester of lactate in muscle in order to reduce plasma lactate produced during exercise (McClelland 2012) and the use lactate utilization for glucose production (Polakof and Soengas 2008; Talarico et al. 2023), reported in situations of energy deficiencies as fasting (Bermejo-Poza et al. 2019; Villalba et al. 2025). Regarding stunning methods, different findings have been reported in tilapia subjected to electronarcosis, where lactate levels were higher in electrical stunning compared to anesthetic treatments (Venturini et al. 2018), though the electrical parameters in that study were considerably higher than those used here. Bermejo-Poza et al. (2021), using comparable stunning methods in optimal rearing temperatures for rainbow trout, found no significant differences in blood lactate concentrations between ice water immersion and electrical shock groups.

### Liver gene expression related with oxidative stress

Oxidative stress, which arises from an imbalance between reactive oxygen species (ROS) production and the organism's ability to detoxify these substances, plays a critical role in both fish welfare and product quality. Antioxidant enzymes such as superoxide dismutase (*sod*), glutathione peroxidase (*gpx*), catalase (*cat*), and glutathione S-transferase (*gst*) are integral in mitigating oxidative stress by reducing ROS levels and are commonly used as markers of oxidative stress (Vrankovic et al. 2021; Zengin 2021). The results related to gene expression of oxidative stress markers are presented in Table 6. It was observed that the gene expression of oxidative stress-related enzymes (*sod*, *gpx*, *cat*, *gst*) was influenced by the season during which stunning occurred, with higher expression levels in summer compared to winter. A significant interaction between stunning method and season was found only for catalase gene expression. In summer, elevated water temperatures increase the metabolic rate of fish due to heightened energy demands (Myrick and Cech 2000), leading to increased ROS production and subsequently activating the antioxidant defense system. This, in turn, results in upregulated expression of antioxidant enzymes (Rich and Maréchal 2010). Dawood et al. (2022) reported that African catfish (*Clarias gariepinus*) showed elevated levels of *sod, gpx* and *cat* liver expression when subjected to heat stress. Additionally, the photoperiod can influence the oxidative stress response, with higher oxidative stress observed in summer. Xu et al. (2022) found that juvenile rainbow trout exposed to longer daylight hours exhibited greater oxidative stress markers compared to those with shorter daylight periods. Regarding *cat* gene expression, a lower expression of TS, ES2 and ES4 groups in summer can be explained by the seasonality and photoperiod. Regarding the stunning methods, different results can be found in literature. Mohammadi Dehcheshmeh et al. (2025) reported that fish *Mesopotamichthys sharpeyi* individuals subjected to electrical treatment exhibited lower levels of *cat* expression in the liver compared to suffocated but Gao et al. (2014) found that electronarcosis induced oxidative stress in the liver of crucian carp (*Carassius carassius*) with higher levels of *cat* and *sod*. Literature findings, alongs with the absence of a higher generalized antioxidant enzymes expression in electric shock groups in the results, makes a definitive explanation for the elevated cat expression in liver of ES2 group in summer unclear.

**Table 6:** Study of oxidative stress in liver through gene expression of rainbow trout subjected to different stunning methods in different season

Parameter		TS	ES2	ES4	Mean	p-value		
						Season	Stunning	Se x St
*sod* gene expression	Winter	1.00 ± 0.10	1.37 ± 0.20	1.38 ± 0.13	1.24 ± 0.04	< 0.0001	0.1225	0.1330
	Summer	24.3 ± 5.30	33.3 ± 5.30	21.9 ± 1.79	26.7 ± 2.24			
	Mean	12.6 ± 11.6	17.3 ± 16.0	11.6 ± 10.3				
*gpx* gene expression	Winter	1.00 ± 0.09	1.21 ± 0.22	1.25 ± 0.13	1.15 ± 0.09	< 0.0001	0.7105	0.9255
	Summer	5.83 ± 0.58	6.32 ± 0.78	6.55 ± 1.04	6.23 ± 0.46			
	Mean	3.41 ± 2.41	3.77 ± 2.55	3.90 ± 2.65				
*catalase* gene expression	Winter	1.00 ± 0.084^d^	1.29 ± 0.24^ cd^	1.24 ± 0.13^d^	1.17 ± 0.09	< 0.0001	< 0.0001	0.0020
	Summer	1.90 ± 0.11^c^	3.36 ± 0.22^a^	2.63 ± 0.12^b^	2.63 ± 0.13			
	Mean	1.45 ± 0.45	2.32 ± 1.04	1.94 ± 0.69				
*gst* gene expression	Winter	1.00 ± 0.10	1.41 ± 0.37	1.38 ± 0.17	1.27 ± 0.14	< 0.0001	0.4832	0.5268
	Summer	42.9 ± 5.01	53.3 ± 8.40	45.9 ± 5.78	47.1 ± 3.79			
	Mean	21.5 ± 20.5	27.3 ± 25.9	23.6 ± 22.3				

*sod *Superoxide dismutase, *gpx* Glutathione peroxidase, *gst* Glutathione-S-transferase, *Se* Season, *St* Stunning, *TS* Thermal shock by ice water immersion, *ES2* Electric shock at 200 mA for 2 s, *ES4* Electric shock at 400 mA for 0.5 s and 200 mA for 1.5 s. Data are presented as mean ± SEM. Different letters indicate significant differences between groups (*p* < 0.05)

## Conclusions

This study underscores the impact of stunning techniques and seasonality on the welfare of rainbow trout, focusing on the physiological stress response through key parameters such as cortisol and corticoid receptor gene expression, skin color, lipid metabolism, carbohydrate utilization, and oxidative stress. Seasonal variations play a crucial role in shaping the physiological state of the fish, particularly in metabolic processes. Elevated temperatures in summer increase the metabolic rate of rainbow trout, leading to enhanced mobilization of lipids and carbohydrates. These metabolic changes are a direct response to increased energy demands and the intensified stress response associated with higher environmental temperatures. The accelerated metabolic activity during summer also exacerbates oxidative stress, as evidenced by higher gene expression levels of antioxidant enzymes, which function to mitigate the oxidative damage caused by increased ROS. Consequently, the stress response triggered by the stunning method may be less pronounced in summer, yet distinct differences remain between the stunning techniques. Notably, higher cortisol levels are observed with thermal shock compared to electrical shock in combination with thermal shock in winter, and variations in skin color and glucose concentration also emerge, with lower blood lactate levels seen in the electrical shock groups, especially at reduced intensities during summer. These findings indicate potential differences in stress responses between stunning techniques, suggesting that combining electrical stunning with thermal shock may provide welfare benefits during stunning, as this method appears to reduce stress more effectively than thermal shock alone. One of the main limitations of this study is the absence of a proper control group in both seasons, which prevents a direct assessment of the baseline physiological state of rainbow trout and, therefore, only allows for understanding the difference impact between stunning methods applied in each season. This limitation makes it more challenging to fully isolate the effects of stunning techniques and environmental factors from natural physiological variations.

## Data Availability

The datasets used or analyzed during the current study are available from the corresponding author on reasonable request.
